# Long-term evaluation of TTK Chitra™ heart valve prosthesis — a retrospective-prospective cohort study

**DOI:** 10.1007/s12055-022-01437-9

**Published:** 2022-12-03

**Authors:** Praveen Kerala Varma, Maniyal Vijayakumar, Gopichettipalayam Subbaratnam Bhuvaneshwar, Adarsh Syla Kumar, Neethu Krishna

**Affiliations:** 1 Department of Cardiovascular and Thoracic Surgery, Amrita Institute of Medical Sciences and Research Centre, Amrita Vishwa Vidyapeetham (Amrita University), Kochi, India; 2 Department of Cardiology, Amrita Institute of Medical Sciences and Research Centre, Amrita Vishwa Vidyapeetham (Amrita University), Kochi, India; 3 Thiruvananthapuram, Kerala India

**Keywords:** TTK Chitra™ heart valve (TTKCHV), Mechanical heart valve, Rheumatic heart disease, Tilting disc valve, Long-term outcomes

## Abstract

**Purpose:**

The TTK Chitra™ heart valve has more than 1,40,000 implantations so far, but no long-term data has been published. This study aims to provide long-term results of the valve.

**Methodology:**

A cohort of 476 patients with implantations from January 2006 to December 2018 were followed up prospectively consisting of 104 aortic valve replacement (AVR), 87 double valve replacement (DVR), and 285 mitral valve replacement (MVR) patients. Total follow-up was 4079 patient-years (py) (AVR = 983, MVR = 2392, DVR = 704), being 96% complete.

**Results:**

The results showed that actuarial survival at 15 years was 82.3% for AVR, 60.7% for MVR, and 52.2% for DVR. Freedom from all valve-related mortality and morbidity at 15 years was 73.8%, 64.8%, and 61.9% for AVR, MVR, and DVR, respectively. There was one instance of structural failure of valve disc leading to severe valvar regurgitation. Valve thrombosis incidence was 1 in AVR (0.1%/py), 6 in MVR (0.25%/py), and 1 in DVR (0.14%/py). Thrombo-embolic episodes occurred in 50 patients (AVR = 7 patients at 0.7%/py; MVR = 36 patients at 1.5%/py; DVR = 7 patients at 0.99%/py) and major hemorrhage (bleeding) in 24 patients (AVR = 0.61%/py; MVR = 0.5%/py; and DVR = 0.85%/py). The linearized rates of adverse events in this study were found to be lower than earlier published results.

**Conclusion:**

The results highlight the continued safety and performance of the TTK Chitra™ heart valve (TTKCHV) in the long term at 15 years.

**Supplementary Information:**

The online version contains supplementary material available at 10.1007/s12055-022-01437-9.

## Introduction

Rheumatic heart disease (RHD) is one of the common indications for heart-valve surgery in India, unlike in developed countries, where degenerative diseases predominate [1, 2]. The overall prevalence of RHD is about 1.5–2/1000 in all age groups. This suggests that there are about 2.0 to 2.5 million patients of RHD in India [1]. The majority of the patients are young and belong to the low socio-economic group with their diseased valves unsuitable for repair [3]. Artificial heart valves have given a new lease of life to patients with valvular disease, substantially improving their quality of life and survival durations.

All artificial heart valves implanted in India were imported until 1990–1991. TTK Chitra™ heart valve (TTKCHV), developed in the late 1980s at the Sree Chitra Tirunal Institute for Medical Sciences and Technology, Thiruvananthapuram, Kerala, aimed to address the need for low-cost mechanical heart valve prosthesis [4]. The new model underwent multi-center clinical trials from December 1990 to February 1995 [5] and has been manufactured and marketed by TTK Healthcare Limited (India) since then. So far, 1,40,000 implantations across 350 major centers in India have been carried out. Several clinical studies published over the last 25 years show the valve to be good and comparable to imported mechanical valve models [5–7]; however, there are no detailed reports of the long-term data (with more than 10-year follow-up) regarding survival, valve-related complication rates, freedom from valve-related events, and improvement in New York Heart Association (NYHA) functional class.

This study was designed to provide a detailed long-term evaluation of the clinical and hemodynamic performance of TTKCHV in a large cohort. Since the turn of the century, clinical laboratory testing services have improved with better test quality at reduced costs. Combined with better patient awareness, it is expected that patient compliance to anticoagulation therapy would have improved. Consequently, this study expects to highlight the changes in the long-term survival of heart valve patients and valve-related complications.

## Materials and methods

### Study design

This study was a retrospective-prospective, single-center, single-arm observational study that included all 476 patients implanted with TTKCHV from 1 January 2006 to 31 December 2018 at a tertiary university hospital in South India. Double valve replacements (DVR), where one valve was different, were excluded. The study was conducted in full conformance with the principles of the “Declaration of Helsinki” (as amended at the 56th World Medical Association (WMA) General Assembly, Tokyo, Japan, 2008). Institutional ethics committee approval was obtained before initiating the study.

All living patients or their close relatives were contacted by mail/phone or approached directly to enrol for a clinical follow-up visit. At the time of patient contact, if it was learned that the patient had died, the reasons for death were obtained to the best possible extent and recorded. Despite considerable efforts, patients, their relatives, or friends who could not be traced or contacted were considered “lost to follow-up.”

#### Retrospective part

The medical records of all study patients were reviewed in detail to gather preoperative, perioperative, and follow-up data such as demographic information, medical history, co-existing medical conditions, previous cardiovascular surgeries, NYHA functional class, blood investigation details, date of surgery, type and size of the TTKCHV implanted, concomitant surgeries, and early postoperative complications.

#### Prospective part

Informed consent was obtained from all patients who agreed to a one-time detailed follow-up and were physically examined by one of the clinical investigators to assess the NYHA functional class and hemodynamic performance. Coagulation profile, hemolysis, complete blood count, plasma hemoglobin, and serum lactate dehydrogenase were obtained by suitable laboratory studies. All events that occurred in patients post valve implantation were recorded. All echocardiography examinations were performed by a senior echo-technician and cross checked by the second author. Patients who were reluctant to attend the follow-up clinic due to personal reasons or the prevailing coronavirus infectious disease (COVID)-19 pandemic were contacted over phone and their clinical status was collected to the extent feasible using a questionnaire. In case of death, all available information, including any possible relationship to the device, was collected from hospital records or close relatives. Lost to follow-up patients’ data from the hospital records were gathered up to their last follow-up and were included in the study. 31 January 2022 was the closing date of the study for the purposes of statistical analysis.

#### Surgical procedure

Cardiopulmonary bypass was established through a midline sternotomy using a membrane oxygenator and moderate hypothermia (26 to 32 °C). Cold blood cardioplegia was used for myocardial protection. After debriding the valve annulus, the prosthesis was secured with interrupted sutures of 2–0 Dacron reinforced with Teflon pledgets using the everting technique, thus inserting the prosthesis in an intra-annular position.

### Study endpoints

The objective of this clinical follow-up study was to determine the long-term (> 10 years) safety and performance of the TTKCH valve in this center. The study endpoints defined in this study plan were therefore as follows:

#### Primary endpoints

- Safety:
  (i) Structural-dysfunction of the valve.
  (ii) Non-structural dysfunction as measured by the linearized rates of late incidence of valve-related adverse events consisting of:
    (a) Thrombosis, (b) thromboembolism, (c) major hemorrhage, (d) major paravalvular leak, (e) hemolysis, and (f) endocarditis
- Performance:
  (i) Long-term total survival and event-free survival at 5, 10, and 15 years
  (ii) Quality of life improvement as shown by improvements in NYHA classification
  (iii) Valve gradients and effective orifice areas (EOA) of the valve measured by Doppler echocardiography.

#### Secondary endpoints

All non-valve-related serious adverse events.

### Data reporting and analysis

The latest guidelines of the Society of Thoracic Surgeons and the American Association for Thoracic Surgery for reporting the morbidity and mortality after heart valve replacement were followed to the extent feasible [8]. Survival analysis by the Kaplan–Meier method included both early and late events. Death due to unknown causes was considered valve-related mortality. The actuarial curves were calculated by the Kaplan–Meier technique using the R—R Studio statistical analysis package and reported with 95% confidence intervals. The difference in survival between the valve groups was calculated by the log-rank method. In the first analysis, “lost-to-follow-up” patients were censored at the time of their last known follow-up, when they were known to be alive. However, to see the effect of “lost to follow-up” patients, in a second analysis they were all considered as “dead” at their last follow-up date and the difference was statistically analyzed using the log-rank test.

Analysis of variance (ANOVA) was used to identify if any significant difference exists in the age and weight of the three valve replacement groups. Univariate logistic regression analysis using the Cox proportional hazard model on mortality was performed to assess the risks associated with 11 factors: body weight, preoperative presence of atrial fibrillation, congestive heart failure, coronary artery disease, history of infective endocarditis, history of thromboembolism, kidney disease, lung disease, previous valve surgeries, percutaneous mitral valvotomy, and concomitant coronary bypass surgery.

Linearized rates were expressed as percent/patient-year (%/py) with ± 1 standard error, which has been computed by the Greenwood formula. Only late events were included in the calculation of linearized rates of complications. Continuous variables were reported as mean ± standard deviation, and categorical variables were represented as percentages. All statistical tests were 2-tailed and a 95% confidence level *p*-value < 0.05 was considered significantly different. These analyses were done using Microsoft Excel version 2013.

A comparison of alive patients who visited for follow-up, patients not willing to visit for follow-up, and patients who were lost to follow-up between three groups — aortic valve replacement (AVR), mitral valve replacement (MVR), and DVR — was done using the chi-square statistics to test the statistical significance (sensitivity analysis). The competing risks regression analysis was done using STATA/IC 15.1 (StataCorp, TX, USA).

## Results

### Study population

In total, 476 patients (MVR = 285, AVR = 104, and DVR = 87) were included in the study. In the prospective part, the first patient attended the follow-up clinic on 15 March 2021 and the last on 12 January 2022. Of the total, 329 (69%) patients were confirmed alive, 128 (27%) confirmed dead, and 19 (4%) lost to follow-up. In the alive group, only 244 (51%) patients attended the follow-up clinic. The status of 85 (18%) alive patients, who could not visit, was obtained through telephonic contact. One hundred four AVR patients had 983 total patient-years (py) of follow-up; 285 MVR patients had 2392 py of follow-up, and 87 DVR patients had 704 py of follow-up. The total follow-up was 4079 py with a mean of 8.6 years; 95% of patients were followed up and the maximum follow-up duration was 15.9 years. The mean age among the three groups combined was 36.5 ± 19 years, the youngest was a 5.5-month-old male MVR patient, and the oldest was a 76-year-old female AVR patient (Table 1). (median 40 years; interquartile range (IQR) 30–48). ANOVA performed on age and weight between the three valve replacement groups showed that there is no significant difference in age (*p* = 0.81) while difference in weight was significant (*p* = 0.02) among the AVR-MVR and MVR-DVR group. In total, 48.9% of patients had comorbidities, 46.4% had co-existing cardiovascular diseases, and 23.1% had previous valve surgeries. The baseline demographic characteristics are summarized in Table 1.

**Table 1:** Study population: demographic characteristics and follow-up

Study details	AVR	MVR	DVR	Total
Total number of patients	104	285	87	476
Male, *n* (%)	81 (78%)	118 (41.4%)	56 (64.4%)	255 (53.6%)
Female, *n* (%)	23 (22%)	167 (58.6%)	31 (35.6%)	221 (46.4%)
Age (mean ± SD) (years)	37.5 ± 18.4	36.7 ± 16.3	40.3 ± 13.4	36.5 ± 19
Age range (min.:max.) in years	(2y:76 y)	(0y 5.5 m:68y)	(18y:70y)	(0y 5.5 m:76y)
Age > 18 (years) (mean ± SD)	43.1 ± 12.4	40.0 ± 10.7	40.3 ± 10.8	40.7 ± 11.2
Age > 18 range (years)	19y–76y	19y–68y	18y–70y	19y–76y
Weight (mean ± SD) (kg)	53.9 ± 15.1	50.3 ± 9.0	53.4 ± 14.3	51.2 ± 15.5
Weight range (min.:max.) (kg)	(11:92)	(3.3:82)	(29.5:115)	(3.3:115)
Weight > 18 y (mean ± SD) (kg)	57.4 ± 10.8	51.2 ± 10.7	53.6 ± 13.3	53.0 ± 11.5
Patient follow-up status				
Lost to follow-up	2	15	2	19
Confirmed dead	12	86	30	128
Confirmed alive — did not attend follow-up clinic	26	48	11	85
Attended the follow-up clinic	64	136	44	244
Early mortality	1	12	4	17
Follow-up details in patients years				
Total possible follow-up (patient-years)	989	2529	731	4251
Actual follow-up (patient-years)	983	2392	704	4079
Percentage follow-up (%)	99%	95%	96%	96%
% followed up	98%	92%	96%	95%
Comorbidities				
Diabetes	17 (16.2)	18 (6.3)	7 (8.0)	42 (8.8)
Endocarditis	8 (7.6)	12 (4.2)	5 (5.7)	25 (5.3)
Thromboembolism	0 (0)	19 (6.7)	0 (0)	19 (4.0)
Hypertension	17 (16.2)	21 (7.4)	5 (5.7)	43 (9.0)
Kidney disease	5 (4.8)	5 (1.8)	2 (2.3)	12 (2.5)
CLD	0 (0)	1 (0.4)	1 (1.1)	2 (0.4)
Lung disease	2 (1.9)	10 (3.5)	4 (4.6)	16 (3.4)
Others	19 (18.1)	48 (16.9)	7 (8.0)	74 (15.5)
Total	68 (65.3)	134 (47.0)	31 (35.6)	233 (48.9)
Co-existing cardiovascular diseases				
Atrial fibrillation	5 (4.8)	93 (32.7)	14 (16.1)	112 (23.5)
CAD	14 (13.3)	15 (5.3)	3 (3.4)	32 (6.7)
CHF	8 (7.7)	11 (3.8)	5 (5.7)	24 (5.0)
Thromboembolism	0 (0)	5 (1.8)	1 (1.1)	6 (1.3)
PVD	0 (0)	2 (0.7)	1 (1.1)	3 (0.6)
Endocarditis	0 (0)	1 (0.4)	2 (2.3)	3 (0.6)
MI (within last 90 days)	0 (0)	0 (0)	1 (1.1)	1 (0.2)
Left atrial thrombus	0 (0)	4 (1.4)	0 (0)	4 (0.8)
Congenital heart disease	0 (0)	1 (0.4)	0 (0)	1 (0.2)
Cardiomyopathy	0 (0)	1 (0.4)	0 (0)	1 (0.2)
Other	15 (14.4)	17 (5.9)	2 (2.3)	34 (7.1)
Total	42 (40.4)	150 (52.6)	29 (33.3)	221 (46.4)
Previous valve operation				
OMV	0 (0)	1 (0.4)	1 (1.1)	2 (0.42)
Percutaneous mitral valvuloplasty	2 (1.9)	72 (25.4)	19 (21.8)	93 (19.5)
Valve operations	6 (5.8)	9 (3.2)	0 (0)	15 (3.2)
Total	8 (7.6)	82 (28.7)	20 (23.0)	110 (23.1)
Etiology				
Mitral stenosis	0	69	20	89
Mitral regurgitation	30	73	19	122
Mitral mixed	3	13	44	60
Aortic stenosis	17	1	4	22
Aortic insufficiency	46	42	43	131
Aortic mixed	39	0	39	78

*AVR* aortic valve replacement, *MVR* mitral valve replacement, *DVR* double valve replacement, *N* number of patients, *y* years, *SD* standard deviation, *CLD* chronic liver disease, *CAD* coronary artery disease, *CHF* congestive heart failure, *PVD* peripheral vascular disease, *MI* myocardial infarction, *OMV* open mitral valvotomy

One hundred ninety-two aortic valves and 371 mitral valve prostheses were used in the study population (Table 2). Ninety-one patients (19.1%) had concomitant procedures (Table 3). One hundred thirty-one patients had aortic valve insufficiency, 22 had aortic valve stenosis, and 78 had mixed aortic valvular lesion. One hundred twenty-two patients had mitral regurgitation, 89 had mitral stenosis, and 60 had mixed mitral valvular lesion. Ninety-three mitral patients had tricuspid regurgitation (TR) in which 45 patients had mild TR, 25 had moderate TR, and 20 had severe TR. One hundred seventy-seven mitral patients had pulmonary artery hypertension (PAH) preoperatively in which 57 patients had severe PAH.

**Table 2:** Prosthesis sizes used

Valve size	Aortic valves used (total, *N* = 192) AVR (104) and DVR (87)		Mitral valves used (total, *N* = 371) MVR (284) and DVR (87)	
	No. of valves	% of total AVR (*N* = 192)	No. of valves	% of total MVR (*N* = 371)
17 mm	12	6.3	0	0
19 mm	59	30.7	0	0
21 mm	46	24	0	0
23 mm	43	22.4	14	3.8
25 mm	28	14.6	80	21.6
27 mm	2	1	113	30.5
29 mm	2	1	81	21.8
31 mm	0	0	83	22.4
33 mm	0	0	0	0

*AVR* aortic valve replacement, *MVR* mitral valve replacement, *DVR* double valve replacement, *N* number of patients

One 17-mm aortic valve implanted in mitral position

**Table 3:** Concomitant surgeries

Concomitant repairs	AVR (*N* = 104)	MVR (*N* = 285)	DVR (*N* = 87)	Total (*N* = 476)
	Number of patients (%)			
Tricuspid/pulmonary valve repair	1 (1.0)	24 (8.5)	12 (13.8)	37 (7.8)
Coronary bypass surgery	10 (9.5)	7 (2.5)	2 (2.3)	19 (4.0)
Left atrial thrombosis removal	0 (0)	3 (1.1)	2 (2.3)	5 (1.1)
ASD/VSD closure or any Septal repair	2 (1.9)	2 (0.7)	0 (0)	4 (0.8)
Aortic valve/annulus repair	0 (0)	2 (0.7)	0 (0)	2 (0.4)
Left atrial appendage removal	0 (0)	3 (1.1)	0 (0)	3 (0.6)
Mitral valve/annulus repair	2 (1.9)	0 (0)	0 (0)	2 (0.4)
Open mitral valvuloplasty	1 (1.0)	0 (0)	0 (0)	1 (0.2)
Aortic root enlargement	1 (1.0)	0 (0)	0 (0)	1 (0.2)
Others	8 (7.7)	6 (2.1)	2 (2.3)	17 (3.6)
Total	25 (23.8)	47 (16.5)	18 (20.7)	91 (19.1)

*AVR* aortic valve replacement, *MVR* mitral valve replacement, *DVR* double valve replacement, *N* number of patients

### Early postoperative complications

Nineteen patients (3.9%) (4 (21.1%) AVR, 10 (52.6%) MVR, and 5 (26.3%) DVR) had early postoperative serious complications. Six patients had pericardial effusion, three had thrombo-embolic events, one had major bleeding event, two had various infections other than valvular endocarditis, two had arrhythmia, and one had congestive heart failure. Three patients had other complications such as pneumothorax (1 patient) and pacemaker implantation (2 patients). One patient with a history of active valve endocarditis continued to have endocarditis post-surgery.

### Mortality

#### Procedural mortality

Procedural mortality (defined as death within 30 days of procedure or discharge from hospital after surgery, whichever is later)[9] occurred in 17 (3.6%) patients (AVR = 1 (0.96%), MVR = 12 (4.2%), DVR = 4 (4.6%)). Five patients died of various infections other than valvular endocarditis, three patients due to congestive heart failure, two patients each due to arrhythmia, multi-organ dysfunction, and sudden cardiac arrest, and one patient each due to cardio-respiratory failure, acute intracerebral hemorrhage, and renal failure.

#### Late mortality

Late deaths occurred in 111 patients (2.7% py) consisting of 11 AVR (1.1% py), 76 MVR (3.2% py), and 24 DVR (3.4% py) patients (Table 4). Twenty-six late deaths were related to valve complications (16 thromboembolism, six hemorrhage, two paravalvular leaks, one valve thrombosis, and one valve endocarditis). Twenty-two unexplained deaths, where the cause could not be established, were considered as valve-related mortality for analysis. Overall, valve-related mortality was 1.17%/py (AVR = 4 cases at 0.4%/py, MVR = 36 cases at 1.5%/py, DVR 8 cases at 1.14%/py). Thirty-five late deaths were due to cardiac complications (2 AVR, 24 MVR, and 9 DVR) and 28 were due to due to other non-valve/non-cardiac-related causes.

**Table 4:** Causes of late death

	Reason for late death	AVR	MVR	DVR	Total
		Number of deaths			
Cardiac related	Cardiac related (total)	2	24	9	35
	Congestive heart failure	0	6	2	8
	Myocardial infarction	0	5	4	9
	Sudden cardiac death	2	12	3	17
	Others (valve conduit procedure)	0	1	0	1
Valve related	Valve related (total)	4	36	8	48
	Valve thrombosis	0	1	0	1
	Major thromboembolism	0	14	2	16
	Major bleeding	1	3	2	6
	Major paravalvular leak	0	1	1	2
	Valve endocarditis	0	1	0	1
	Sudden unexplained deaths	3	16	3	22
Other causes	Other (total)	5	16	7	28
	Hepatic failure	0	6	1	7
	Sepsis	0	1	4	5
	Renal failure	1	2	1	4
	COVID-19	1	0	1	2
	Meningioma	0	1	0	1
	Malignancy	1	2	0	3
	Road traffic accident	0	2	0	2
	Suicide	1	1	0	2
	Electric shock	0	1	0	1
	Total	11	76	24	111
	Linearized rate**s (%/py)**	1.1	3.2	3.4	2.7

*AVR* aortic valve replacement, *MVR* mitral valve replacement, *DVR* double valve replacement, *N* number of patients, *py* patient-year, *COVID* coronavirus infectious disease

### Valve-related complications

One hundred thirty-four valve-related serious adverse events occurred; 7 early events and 127 late events (Table 5). A comparison between the linearized rates of these events with the objective performance criteria (OPC) specified in International Organisation for Standardisation (ISO) 5840–2:2021 Annexure I [9–11] showed that the rate of events was lower and well within the limits of the international standard for mechanical heart valves.

(i) ***Structural deterioration***: A 21-year-old male patient, who underwent MVR with 27 mm TTKCHV in 2007, presented with shortness of breath in 2016. Echocardiography showed severe mitral regurgitation and the patient underwent reoperation to replace the mitral valve prosthesis with the same size TTKCHV. The explanted valve had disc deterioration with cracks in the disc, which caused the regurgitation. The incident occurred after 9 years and 5 months of implantation.
(ii) ***Reoperation***: Seven patients underwent reoperation (1 AVR at 0.1%/py, 5 MVR at 0.21%/py, and 1 DVR at 0.14%/py) due to non-structural issues. Five reoperations were due to valve endocarditis, and one each was due to paravalvular leak and pannus overgrowth. Before the end of 1 year, three MVR patients underwent reoperation, where two patients were operated for infective endocarditis and the third patient underwent a DVR to replace the aortic valve due to severe aortic valve regurgitation and a reoperation at the mitral position owing to severe mitral paravalvular leak. At the end of 5, 7, and 13 years, one patient each had re-replacement surgeries due to endocarditis (1 DVR, 1 AVR, and 1 MVR). At the end of 10 years, one MVR patient underwent a reoperation for pannus overgrowth.
(iii) ***Valve thrombosis***: There were eight cases of valve thrombosis (1 AVR at 0.1%/py, 6 MVR at 0.25%/py, and 1 DVR at 0.14%/py) which were thrombolyzed as per institutional practice (reoperation is considered only in failed thrombolysis or in patients presenting with cardiogenic shock and pulmonary edema). Seven patients responded well to thrombolytic therapy with streptokinase; six patients recovered without any residual morbidity, but one patient (MVR) died after 2 years and 11 months due to myocardial infarction. One MVR patient was in NYHA class IV at admission and had thrombolysis with streptokinase, but the patient suffered hypoxic brain damage that led to mortality. Five MVR patients had suboptimal international normalized ratio (INR) values at the time of the event.
(iv) ***Embolism***: There were fifty late episodes of thromboembolism (AVR = 7 cases at 0.7%/py, MVR = 36 cases at 1.5%/py, DVR = 7 cases at 0.99%/py). Forty-seven embolic events were neurologic, while three were peripheral. The majority of them had low INR and responded to adjustment of anticoagulant dosage. Sixteen patients (14 MVR, 2 DVR) died due to thrombo-embolic events.
(v) ***Major bleeding***: There were twenty-four late major bleeding events (6 AVR at 0.61%/py, 12 MVR at 0.5%/py, and 6 DVR at 0.85%/py) with six deaths (1 AVR, 3 MVR, and 2 DVR). Nine patients (6 MVR and 3 DVR) had INR levels out of the prescribed range and were treated by adjusting the anticoagulant dosage.
(vi) ***Endocarditis****:* Eleven patients (2 AVR at 0.2%/py, 6 MVR at 0.29%/py, 3 DVR at 0.42%/py) had infective endocarditis. One patient had active endocarditis preoperatively and continued to have it post-surgery; antibiotic treatment was provided and fully resolved. Five patients were re-operated and replaced with a new valve, five patients were medically managed, and one patient died due to septic shock.
(vii) ***Hemolysis***: The plasma hemoglobin (Hb), serum lactate dehydrogenase (LDH), and reticulocyte counts were used as markers for hemolysis. The plasma Hb level (mg/L) was: in AVR 55.69 ± 28.85; in MVR 54.93 ± 20.15; and in DVR 55.84 ± 24.98. Serum LDH (U/L): in AVR 298.11 ± 66.77; in MVR 369.2 ± 122.58; and in DVR 343.7 ± 160.69. Reticulocyte count (%): in AVR 1.09 ± 0.47; in MVR 1.08 ± 0.47; and in DVR 1.23 ± 0.61. The patients did not have clinically significant hemolysis or anemia.

**Table 5:** Details of valve-related complications

Valve-related serious adverse events	AVR				MVR				DVR			Total VR complications	Linearized rates (%/py)	
	Number of incidences													
	Early		Late		Early	Late		Early		Late				
Valve disc damage	0		0		0	1		0		0	1		0.025 ± 0.025	
Inappropriate valve size*	0		1		0	0		0		0	1		0.025 ± 0.025	
Major bleeding	1		6		0	12		1		6	26		0.64 ± 0.12	
Major paravalvular leak	0		3		0	1		0		2	6		0.15 ± 0.06	
Major thromboembolism — neurologic	0		7		4	33		0		7	51		1.32 ± 0.18	
Major thromboembolism — peripheral	0		0		0	3		0		0	3			
Valve endocarditis	0		2		1	7		0		3	13		0.32 ± 0.08	
Valve thrombosis	0		1		0	6		0		1	8		0.2 ± 0.07	
Other non-structural	0		3		0	17		0		3	23		0.56 ± 0.12	
Pannus overgrowth	0		0		0	1		0		1	2		0.05 ± 0.03	
Total	1		23		5	81		1		23	134			
Linearized rate of late complications compared with OPC from ISO 5840														
Late complications		Aortic (% py) ± Std dev					Mitral (% py) ± Std dev					Double (% py) ± Std dev		
		Std OPC		TTKCHV			Std OPC		TTKCHV			Std OPC**		TTKCHV
Valve thrombosis		0.1		0.10 ± 0.10			0.2		0.25 ± 0.10			0.2		0.14 ± 0.14
Thromboembolism		1.6		0.71 ± 0.26			2.2		1.5 ± 0.25			2.7		0.99 ± 0.38
Major hemorrhage		1.6		0.61 ± 0.24			1.4		0.5 ± 0.14			2.1		0.85 ± 0.35
Major paravalvular leak		0.3		0.30 ± 0.18			0.5		0.04 ± 0.04			0.6		0.28 ± 0.20
Endocarditis		0.3		0.20 ± 0.14			0.3		0.29 ± 0.11			0.4		0.43 ± 0.07

*AVR* aortic valve replacement, *MVR* mitral valve replacement, *DVR* double valve replacement, *N* number of patients, *VR* valve related, *py* patient-year, *OPC* objective performance criteria, *ISO* International Organization for Standardization, *Std dev* standard deviation, *TTKCHV* TTK Chitra™ heart valve. *Inappropriate valve size indicates PPM observed in a pediatric patient after 6 years of implantation. **OPC criteria for double valve replacements were derived from the OPC values for aortic and mitral as the Sqrt (av^2^ + mv^2^)

### Survival analysis

**Fig. 1:**
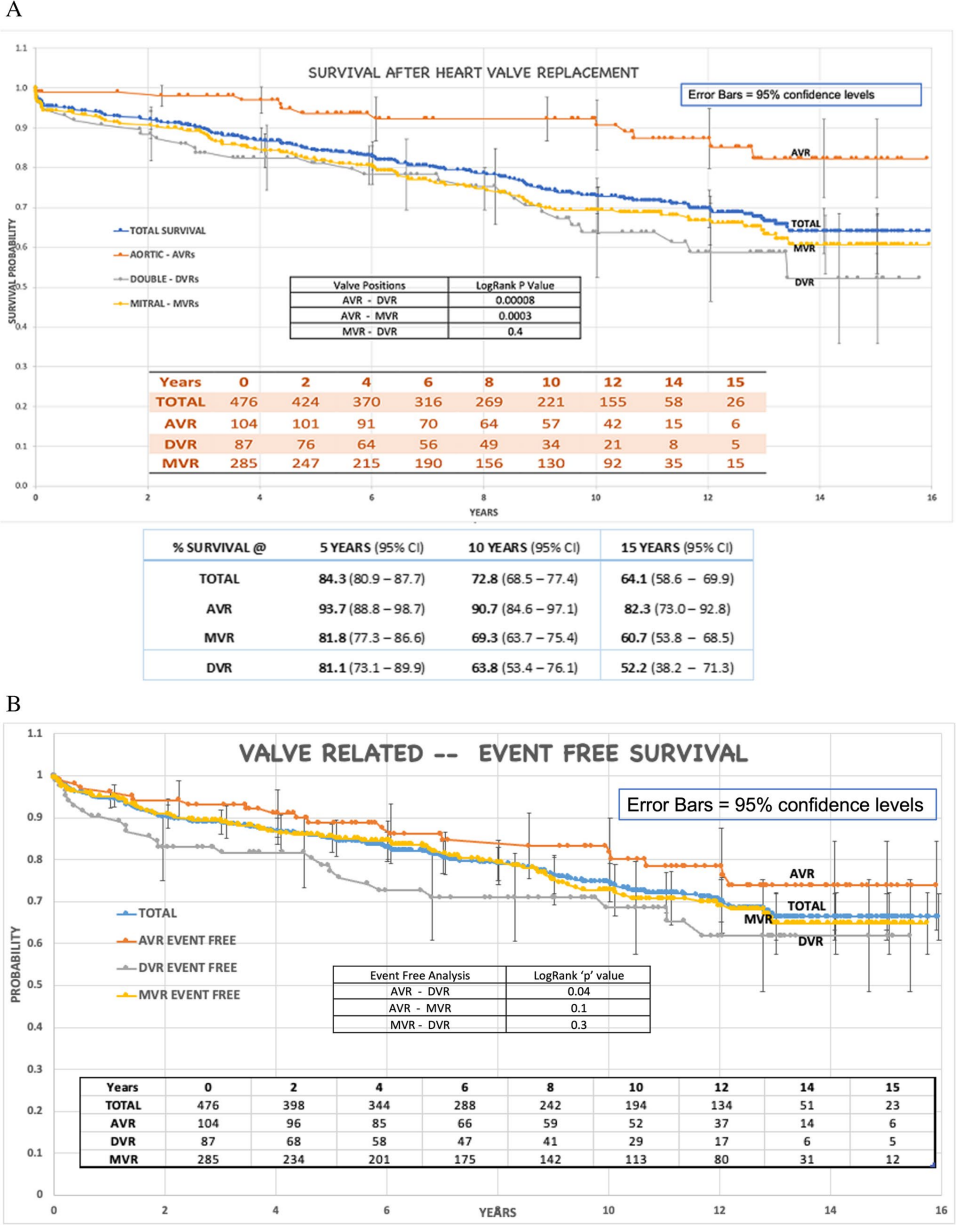
**A** Total actuarial survival. **B** Event-free survival

The actuarial survival rates for TTKCHV patients at 5 years, 10 years, and 15 years is 93.7%, 90.7%, and 82.3% for AVRs; 81.8%, 69.3%, and 60.7% for MVR; and 81.1%, 63.8%, and 52.2% for DVR (Fig. 1A). The difference in survival between the AVR-DVR and AVR-MVR groups (log-rank method) was significant at *p* = 0.00008 and *p* = 0.0003, respectively, but no significant difference exists between DVR and MVR (*p* = 0.4) (Fig. 1A). When the lost to follow-up patients were also considered as “dead,” the log-rank test showed no significant difference between the two pairs of survival curves for each valve group (*p* = 0.7, 0.3, and 0.8 for AVR, MVR, and DVR groups, respectively).

The result of Cox regression analysis revealed that preoperative presence of atrial fibrillation, congestive heart failure, coronary artery disease, infective endocarditis, thromboembolism, kidney disease, lung disease, previous valve surgeries, and concomitant coronary bypass surgery were not significant for survival in any of the three groups. In MVR survival, the covariates previous percutaneous mitral valvotomy and adult patient weight showed statistical significance. Percutaneous mitral valvotomy showed an association with low mortality (*p* = 0.01, hazard ratio 0.16), while patient weight outside the mean weight range showed an increased risk (*p* = 0.013, hazard ratio 1.73) (supplemental file, Table 1) compared to AVR.

### Event-free analysis

The event-free actuarial survival rates for TTKCHV at 5 years, 10 years, and 15 years from serious valve-related morbidity and mortality are 88.8%, 81.7%, and 73.8% for AVR; 85.5%, 72.7%, and 64.8% for MVR; and 77.2%, 68.6%, and 61.9% for DVR (Fig. 1B). The difference between the AVR-DVR was significant at *p* = 0.04 (log-rank method), but no significant difference existed between DVR-MVR and AVR-MVR at *p* = 0.3 and 0.1, respectively.

### Sensitivity analysis

Sensitivity analysis by including 4% lost to follow-up showed that the three groups was equally distributed and there was no significant variation between the groups with a *p*-value of 0.243 inferring that it will not affect the results of the study.

### Competing risks regression

In competing risks regression analysis, the MVR group was compared with the AVR group on valve-related deaths with non-valve-related deaths as a competing risk factor. The MVR group has statistical significance with a *p*-value 0.019 and sub-distribution hazard ratio (SHR) 3.37 (1.22–9.33) when compared to the AVR group. There was no significant difference when DVR was compared with AVR and MVR was compared with DVR.

### Valve performance by echocardiography

Peak and mean gradient measurements for the 244 patients showed that the hemodynamic performance of TTKCHV is good in all valve sizes. One patient with a size 17 mm was observed to have high gradients due to severe prosthesis-patient mismatch (PPM) and was planned for a reoperation (Table 6).

**Table 6:** TTKCHV gradients and effective orifice area (EOA) (for 244 out of 476 patients)

Valve size	No. of patients	Peak gradient (mmHg)	Mean gradient (mmHg)	EOA cm^2^
TTKCHV aortic prosthesis				
17 mm	*N* = 6	58.26 ± 31.4	36.6 ± 18.7	0.64 ± 0.34
19 mm	*N* = 26	44.06 ± 15.77	25.86 ± 10.1	1.03 ± 0.18
21 mm	*N* = 27	32.67 ± 12.91	18.1 ± 7.7	1.29 ± 0.43
23 mm	*N* = 27	23.17 ± 6.40	12.76 ± 3.8	1.40 ± 0.26
25 mm	*N* = 18	21.78 ± 11.1	11.7 ± 5.1	1.6 ± 0.46
TTKCHV mitral prosthesis				
23 mm	*N* = 10	15.88 ± 2.73	5.4 ± 1.5	1.19 ± 0.22
25 mm	*N* = 41	13.59 ± 4.76	5.28 ± 2.7	1.44 ± 0.35
27 mm	*N* = 51	12.19 ± 3.79	5.01 ± 2.5	1.52 ± 0.33
29 mm	*N* = 41	11.6 ± 3.76	4.29 ± 1.3	1.75 ± 0.48
31 mm	*N* = 33	11.26 ± 3.43	4.27 ± 1.2	1.58 ± 0.34

*TTKCHV* TTK Chitra™ heart valve, *EOA* effective orifice area

Table 6 provides hemodynamic performance of 244 patients who visited for follow-up and not the entire cohort

### Functional class

**Fig. 2:**
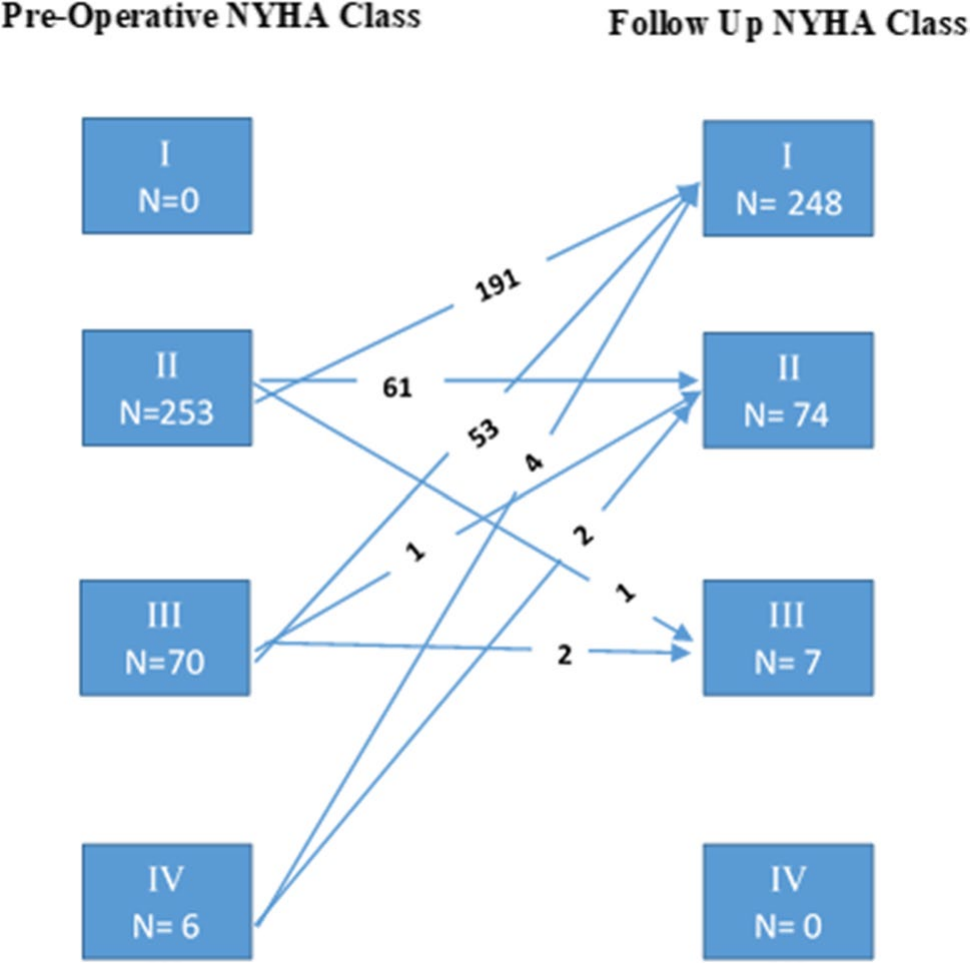
Preoperative and follow-up NYHA class of followed up patients (*N* = 329)

Preoperatively, 363 (76%) patients were in the NYHA class II, 100 (21%) in class III, and 13 (3%) in class IV. Thirty-four percent of class II patients had preoperative comorbidities such as coronary artery disease, congestive heart failure, and atrial fibrillation, while 20% had previous cardiovascular operations. The clinical follow-up assessed that out of the 329 alive patients, 248 (75%) patients moved up to class I, 74 (23%) patients to class II, and 7 (2%) patients to class III post valve replacement. The high disability class IV patients also moved into class II or III postoperatively (Fig. 2).

## Discussion

This study covered 476 patients implanted with TTKCHV with total follow-up of 4079 py and reports the actuarial survival and freedom from valve-related complications at 15 years. A significant outcome of this study was the high survival percentage of 82.2% for AVR patients at 15 years, which was higher than internationally reported for other proven valve designs [12–14].

Three papers have reported follow-up results of TTKCHV up to 10 years. Sankarkumar et al. [5] reported the follow-up of the first multi-center cohort of 306 patients from December 1997 to March 1998 and covered survival at 7 years and linearized incidence of valve-related complications, etc. Muralidharan et al. [6] reported the 10-year outcome of 65 patients (who were part of the same first cohort as in the [5]). Joshi et al. [7] covered 178 valve replacements from January 1993 to December 2003 and reported survival up to 10 years with a total follow-up of 448 py. This study covers a long-term follow-up of 15 years for TTKCHV with 4079 py.

The sensitivity analysis of lost to follow-up patients on the long-term survival showed that there is no statistically significant difference between censoring them at their known last follow-up date or assuming them as “dead” from that point of time. This highlights the robustness of the data in this study.

The ISO standard for surgically implanted heart valve substitutes [10] mentions that OPC are the average linearized complication rates derived based on an analysis of the safety and effectiveness data submitted by manufacturers in pursuit of premarket approval of bioprosthetic and mechanical valves (yielding 38,359 follow-up years) combined with an analysis of literature from 1999 to 2012 (yielding 208,585 follow-up years). Hence, a comparison between the linearized rates of valve-related events with the OPC was performed. The lower linearized rates here highlight the continued safety in the long-term performance of the TTKCHV. The current complication rates for thrombosis, thromboembolism, and bleeding were lower than the rates reported during the first clinical trial of TTKCHV during the 1990s. Data show that the difference in linearized rates of complications was statistically significant (*p* = 0.04).

Cox proportional hazard model analysis showed that preoperative atrial fibrillation and congestive heart failure were not significant to survival, which was similar to the results obtained from the previous multi-center clinical trial results of TTKCHV [2]. The improved availability of better quality clinical laboratory testing, emphasis and management of anticoagulant therapy by clinicians, and enhanced awareness and understanding of patients are perhaps the contributors to the reduced incidences observed in this study (Table 7).

**Table 7:** Comparison of linearized rates

Events	Valve type	Linearized rates (Sankarkumar et al. [5])	Linearized rates (this study)
No. of patients		AVR = 101 MVR = 205	AVR = 104 MVR = 285
All deaths	AVR	3.8 ± 0.9	1.22 ± 0.35
	MVR	7.3 ± 1.0	3.66 ± 0.39
Thrombosis	AVR	0.2 ± 0.2	0.10 ± 0.10
	MVR	1.6 ± 0.5	0.25 ± 0.10
Embolism	AVR	1.6 ± 0.6	0.71 ± 0.26
	MVR	2.4 ± 0.6	1.42 ± 0.24
Bleeding	AVR	0.9 ± 0.5	0.60 ± 0.24
	MVR	0.4 ± 0.2	0.5 ± 0.14
Infective endocarditis	AVR	0.7 ± 0.4	0.20 ± 0.14
	MVR	0.5 ± 0.3	0.29 ± 0.11
VR mortality	AVR	2.3 ± 0.7	0.40 ± 0.20
	MVR	4.0 ± 0.7	1.5 ± 0.25

*AVR* aortic valve replacement, *MVR* mitral valve replacement, *DVR* double valve replacement, *VR* valve related

Another difference noted was the change in the patient profile. The mean age of patients was 28.9 years during the 1990s [2], but now it had increased by almost 8 years to 36.5 years. One reason for this could be the increased use of valve repair techniques and balloon mitral valvotomy leading to delayed replacement with mitral valves. The other major reason could be the general awareness and better quality prophylactic management of children, who are at risk of acquiring rheumatic valve disease and the general decline in the prevalence of RHD in the state of Kerala [15].

Despite improvements in testing and anticoagulant management, nearly 70% (167 patients) who attended the follow-up clinic had INR values outside the recommended control range. In this study group, 132 patients had INR below the target range. One hundred thirty-two patients (20 AVR, 86 MVR, and 26 DVR) who were followed up had INR values below the therapeutic range of 2–3 for AVR and 2.5–3.5 for MVR and DVR. In the AVR group, 6 patients had INR between 1.5 to 2 while 18 had INR between 1.0 and 21.5. The average of low INR for aortic patients was 1.57. In the MVR/DVR group, 47 patients had INR between 2 and 2.5, 35 had between 1.5 and 2, and 29 had between 1 and 1.5. The average low INR value for MVR/DVR group was 1.45 and the lowest recorded INR value was 0.94. Even with these lower levels of anticoagulation in this cohort, the incidence of valve-related complications, particularly valve thrombosis and thromboembolism was below the internationally reported levels. This brings up the question as to whether the current internationally recommended target INR levels which are being followed in India are suitable for Indian patients with TTKCHV. A much larger multi-center study could lead to better clarity on this issue and help determine the most suitable target INR ranges for our patients. Also, the development and wide spread use of a low-cost point-of-care test for INR will greatly help in improving INR monitoring and improve compliance with this critical requirement [16]. Twenty-four late major bleeding events suggest that better control and monitoring of anticoagulation therapy in MVR and DVR patients can reduce the incidence of bleeding events.

There was one late occurrence of structural impairment of the ultra-high molecular weight polyethylene (UHMWPE) plastic leaflet of the valve in this study after 9 years and 5 months of implantation similar to the one reported by Sundaram et al. [17]. The patient had symptoms and sought medical care. The damaged valve was re-replaced with a 27-mm TTKCHV, and the patient was discharged uneventfully. The incidence of structural failure of the TTKCHV continues to be very low but needs to be considered during patient follow-up.

Peak and mean gradient measurements for 244 patients showed that the hemodynamic performance of TTKCHV is comparable to the values reported earlier [17–20] and other well-known mechanical valves (tilting disc and bileaflet) as published by the American Society of Echocardiography [21]. Nagarajan et al. [22] reported comparable hemodynamic performance and gradients across the TTKCHV to other mechanical valves. Namboodiri et al. [23] studied the Doppler echocardiography parameters obtained with the TTKCHV in the mitral position and found that it was comparable to different prosthetic valves in common use. The valve gradients and EOA, as measured by echocardiography, showed consistent performance of TTKCHV in comparison to earlier published results (supplemental file, Table 2). Three of the six patients implanted with 17-mm aortic valve belonged to pediatric population which leads to PPM in these patients.

LDH levels and plasma Hb levels were marginally raised in patients with TTKCHV of all sizes, both in the aortic and mitral positions as in common for all mechanical valves. The patients did not have clinically significant hemolysis or anemia. The hemolytic potential of the TTKCHV was comparable to that of other commercially used mechanical valves [24–26] as seen by comparing with LDH values from those studies.

## Conclusion

The TTKCHV continues to be a safe and effective mechanical heart valve for the replacement of diseased heart valves as evidenced in this long-term clinical study.

## Limitations

This is a single-center study and results are confined to data from one center. Due to the COVID-19 situation, 85 patients were not willing to visit for physical follow-up. Hence, echocardiography and blood study results are reported based on data from 244 patients who visited for follow-up. Pediatric patients were included to provide a detailed and comprehensive outcome and have led to some heterogeneity. This should be considered while interpreting mean age of implantation and survival data. Very low number of left atrial appendage exclusion is another limitation of this study as this could have contributed to an increased incidence of thrombo-embolic events.

## Supplementary Information

Below is the link to the electronic supplementary material.

Supplementary file1 (DOCX 18 KB)
