# Supplementary material for: Long-term evaluation of TTK Chitra™ heart valve prosthesis — a retrospective-prospective cohort study
Source: Indian J Thorac Cardiovasc Surg. 2022 Dec 3;39(1):14–26. doi: 10.1007/s12055-022-01437-9 (PMC9735205; doi:10.1007/s12055-022-01437-9)
Supplement: Supplementary file 1 — Supplementary file1 (DOCX 18 KB) [file 12055_2022_1437_MOESM1_ESM.docx]

**Table 1: Cox regression analysis**

| **Factor** | **Type of surgery** | ***Hazard ratio [exp (coef)]***  **(95% CI)** | **p value** |
| --- | --- | --- | --- |
| AF | AVR | 3.056 (0.382 -24.4) | 0.292 |
|  | MVR | 1.241 (0.793 - 1.942 | 0.344 |
|  | DVR | 1.759 (0.754 - 4.105) | 0.191 |
| Weight | AVR | 0.90 (0.24 -3.32) | 0.875 |
|  | MVR | 1.73 (1.12 -2.69) | 0.013* |
|  | DVR | 1.86 (0.91 – 3.84) | 0.09 |
| CHF | AVR | 1.163 (0.149 -9.052) | 0.885 |
|  | MVR | 0.720 (0.177- 2.931) | 0.647 |
|  | DVR | 1.081 (0.257 -4.554) | 0.916 |
| CAD | AVR | 2.920 (0.778 - 10.96) | 0.112 |
|  | MVR | 2.008 (0.926 - 4.353) | 0.078 |
|  | DVR | 3.340 ( 0.788 -14.16) | 0.102 |
| Kidney disease | AVR | 2.303(0.297-17.88) | 0.425 |
|  | MVR | 1.927 (0.473 -7.851) | 0.36 |
|  | DVR | 1.144 (0.155-8.439) | 0.895 |
| History of IE | AVR | 1.339e-08 (0-Inf) | 0.998 |
|  | MVR | 0.694 (0.170-2.827) | 0.61 |
|  | DVR | 0.970 (0.231-4.086) | 0.967 |
| History of thromboembolism | MVR | 1.461 (0.634-3.365) | 0.373 |
|  | DVR | 2.609 (0.352-19.34) | 0.348 |
| Co-existing lung disease | AVR | 5.827(0.736-46.14) | 0.095 |
|  | MVR | 0.572 (0.141-2.328) | 0.436 |
|  | DVR | 1.758 (0.415 -7.439) | 0.444 |
| Concomitant Coronary bypass surgery | AVR | 1.060 (0.135-8.331) | 0.956 |
|  | MVR | 2.275 (0.833-6.213) | 0.109 |
|  | DVR | 4.933 (0.646-37.65) | 0.124 |
| Previous valve surgeries | AVR | 1.324e-08(0-Inf) | 0.998 |
|  | MVR | 0.367 (0.051-2.638) | 0.319 |
|  | DVR | 3.895e-08(0-Inf) | 0.998 |
| Percutaneous mitral valvotomy | MVR | 0.158 (0.039-0.644) | 0.01* |
|  | DVR | 0.191 (0.026-1.402) | 0.104 |

Significant p value <0.05*

AF: atrial fibrillation, CHF: congestive heart failure, CAD: coronary artery disease, IE: infective endocarditis, AVR: aortic valve replacement, MVR: mitral valve replacement, DVR: double valve replacement, CI: confidence interval

Table 2: In vivo MEAN EOA Values for TTK Chitra Heart valves

| Type | Valve Size (mm) | Study | No: of patients | Mean EOA (previous studies) | Mean EOA (this study) |
| --- | --- | --- | --- | --- | --- |
| Mitral | 25 | Namboodiri et al^21^(2008) | 13 | 1.42± 0.35 | 1.44 ± 0.35 |
|  |  | Sreenivasaetal^18^(2016)* | 48 | 2.7 ± 0.8 |  |
|  | 27 | Namboodiri et al^21^(2008) | 22 | 1.56 ± 0.29 | 1.52 ± 0.33 |
|  |  | Sreenivasaetal^18^(2016)* | 35 | 2.9 ± 0.5 |  |
|  | 29 | Namboodiri et al^21^(2008) | 5 | 1.81 ± 0.59 | 1.75 ± 0.48 |
|  |  | Sreenivasaetal^18^(2016)* | 10 | 3.0 ± 0.6 |  |
| Aortic | 19 | Namboodiri et al^16^(2010) | 12 | 0.91 ± 0.19 | 1.03 ± 0.18 |
|  |  | SaravanaBabu et al^17^(2018) | 8 | 1.35 ± 0.21 |  |
|  |  | Sreenivasaetal^18^(2016) | 7 | 1.59 ± 0.5 |  |
|  | 21 | Namboodiri et al^16^(2010) | 34 | 1.13 ± 0.25 | 1.29 ± 0.43 |
|  |  | Sreenivasaetal^18^(2016) | 18 | 1.8 ± 0.3 |  |
|  |  | SaravanaBabu et al^17^(2018) | 15 | 1.61 ± 0. 19 |  |
|  | 23 | SaravanaBabu et al^17^(2018) | 9 | 2.04 ± 0.14 | 1.4 ± 0.26 |
|  |  | Namboodiri et al^16^(2010) | 26 | 1.49 ± 0.27 |  |
|  |  | Sreenivasa etal^18^(2016) | 16 | 1.9 ±0.5 |  |
|  | 25 | SaravanaBabu et al^17^(2018) | 3 | 2.19 ± 0.13 | 1.6 ± 0.46 |
|  |  | Namboodiri et al^16^(2010) | 19 | 1.93 ± 0.39 |  |

EOA: Effective Orifice Area, *Measured using pressure halftime
